# T1-weighted fast fluid-attenuated inversion-recovery sequence (T1-FFLAIR) enables the visualization and quantification of fetal brain myelination *in utero*

**DOI:** 10.1007/s00330-023-10401-z

**Published:** 2023-11-29

**Authors:** Ruxandra-Iulia Milos, Victor Schmidbauer, Martin L. Watzenboeck, Friedrich Stuhr, Gerlinde Maria Gruber, Christian Mitter, Gregor O. Dovjak, Marija Milković-Periša, Ivica Kostovic, Nataša Jovanov-Milošević, Gregor Kasprian, Daniela Prayer

**Affiliations:** 1 Department of Biomedical Imaging and Image-Guided Therapy, Medical University of Vienna, Waehringer Guertel 18-20, 1090 Vienna, Austria; 2 Department of Anatomy and Biomechanics, Karl Landsteiner University of Health Sciences, 3500 Krems, Austria; 3 Department of Pathology and Cytology, University Hospital Centre Zagreb, Petrova 13, 10000 Zagreb, Croatia; 4 Croatian Institute for Brain Research, School of Medicine, University of Zagreb, Zagreb, Croatia; 5 Department of Biology, School of Medicine, University of Zagreb, Zagreb, Croatia

**Keywords:** Fetal imaging, Brain, Magnetic resonance imaging, White matter

## Abstract

**Objectives:**

To investigate the advantage of T1-weighted fast fluid-attenuated inversion-recovery MRI sequence without (T1-FFLAIR) and with compressed sensing technology (T1-FFLAIR-CS), which shows improved T1-weighted contrast, over standard used T1-weighted fast field echo (T1-FFE) sequence for the assessment of fetal myelination.

**Materials and methods:**

This retrospective single-center study included 115 consecutive fetal brain MRI examinations (63 axial and 76 coronal, mean gestational age (GA) 28.56 ± 5.23 weeks, range 19–39 weeks). Two raters, blinded to GA, qualitatively assessed a fetal myelin total score (MTS) on each T1-weighted sequence at five brain regions (medulla oblongata, pons, mesencephalon, thalamus, central region). One rater performed region-of-interest quantitative analysis (*n* = 61) at the same five brain regions. Pearson correlation analysis was applied for correlation of MTS and of the signal intensity ratios (relative to muscle) with GA on each T1-weighted sequence. Fetal MRI–based results were compared with myelination patterns of postmortem fetal human brains (*n* = 46; GA 18 to 42), processed by histological and immunohistochemical analysis.

**Results:**

MTS positively correlated with GA on all three sequences (all *r* between 0.802 and 0.908). The signal intensity ratios measured at the five brain regions correlated best with GA on T1-FFLAIR (*r* between 0.583 and 0.785). T1-FFLAIR demonstrated significantly better correlations with GA than T1-FFE for both qualitative and quantitative analysis (all *p* < 0.05). Furthermore, T1-FFLAIR enabled the best visualization of myelinated brain structures when compared to histology.

**Conclusion:**

T1-FFLAIR outperforms the standard T1-FFE sequence in the visualization of fetal brain myelination, as demonstrated by qualitative and quantitative methods.

**Clinical relevance statement:**

T1-weighted fast fluid-attenuated inversion-recovery sequence (T1-FFLAIR) provided best visualization and quantification of myelination in utero that, in addition to the relatively short acquisition time, makes feasible its routine application in fetal MRI for the assessment of brain myelination.

**Key Points:**

*• So far, the assessment of fetal myelination in utero was limited due to the insufficient contrast.*

*• T1-weighted fast fluid-attenuated inversion-recovery sequence allows a qualitative and quantitative assessment of fetal brain myelination.*

*• T1-weighted fast fluid-attenuated inversion-recovery sequence outperforms the standard used T1-weighted sequence for visualization and quantification of myelination in utero.*

**Graphical abstract:**

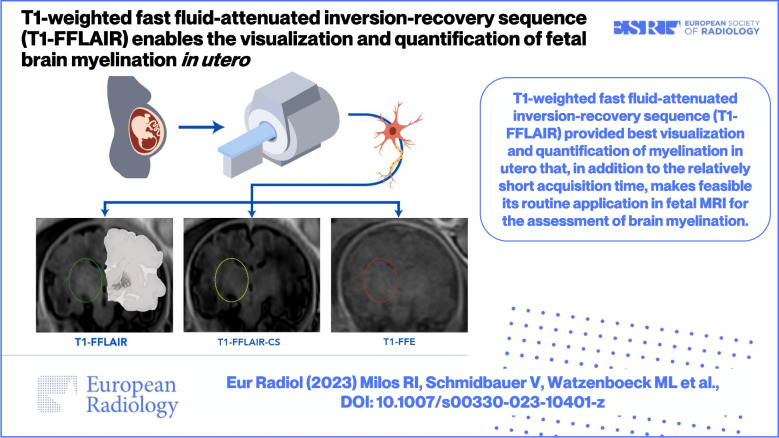

**Supplementary information:**

The online version contains supplementary material available at 10.1007/s00330-023-10401-z.

## Introduction

Myelination represents one of the main processes of white matter maturation. Although predominantly a postnatal process, myelination begins prenatally around gestational age (GA) of 18 weeks in the brainstem, progressing systematically to cranial and rostral brain regions [1]. On magnetic resonance imaging (MRI), myelination appears hyperintense to surrounding tissue on conventional T1-weighted sequences, due to T1 shortening induced by the components of the developing myelin sheaths (cholesterol, glycolipids, and proteins) [2]. MRI studies performed on preterm brains showed that, before GA of 28–30 weeks, myelination-corresponding hyperintensity was demonstrated in several gray matter nuclei and white matter tracts of the brainstem, ventrolateral thalamus, dentate nucleus, and vermis cerebelli, as well as cerebellar peduncles [3]. After GA36, myelin appeared in the lateral part of the internal capsule, in central parts of the corona radiata, and in the cortex bordering the central sulcus [3, 4].

Currently, data on the evaluation of fetal brain myelination in utero are scarce. The image quality of routinely used T1-weighted sequences in fetal MRI is limited due to motion artifacts and insufficient contrast. However, inversion-recovery techniques designed to null the signal from the cerebrospinal fluid (CSF) enhance T1-weighted contrast [5–8]. Furthermore, the new compressed sensing technology now enables a significantly accelerated image acquisition [9], thus shortening the scan time and movement artifacts.

We hypothesized that acquiring a single-shot high-resolution TSE**-**based T1-weighted fast FLAIR MRI sequence [5] without (T1-FFLAIR) and with compressed sensing (T1-FFLAIR-CS) would facilitate the visualization of myelination due to an increase in contrast [5] and improved detailed anatomic delineation [6].

## Methods

### Study cohort

The local ethics commission approved the protocol for this retrospective single-center study (EK Nr: 1695/2021). Written, informed consent for the scientific use of images was obtained before the MRI study. No sedatives were given during examinations. Gestational age was calculated based on the last menstrual period or, in equivocal cases, on the results of fetal ultrasound biometry.

**Fig. 1:**
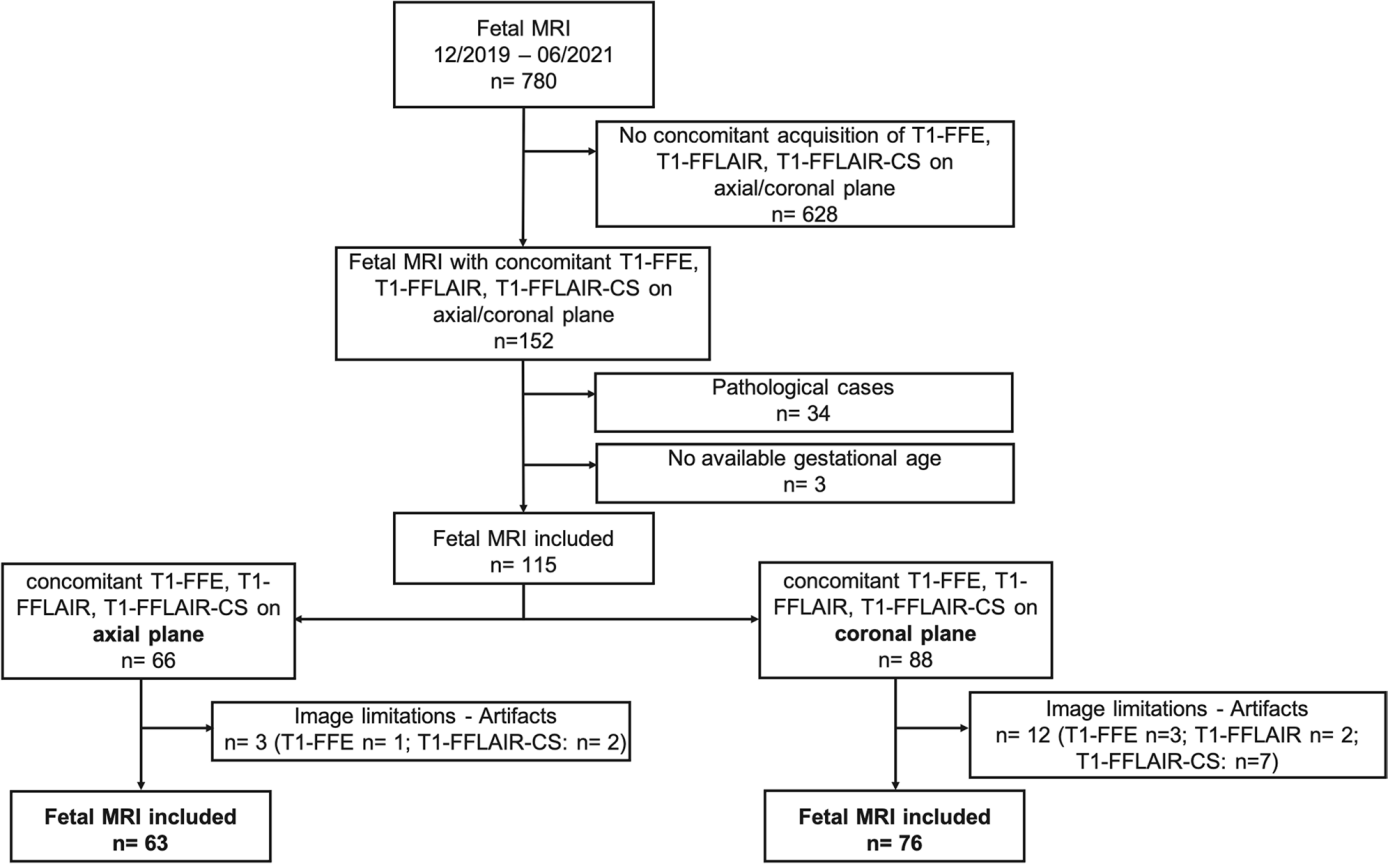
Flowchart of the study design. The inclusion and exclusion criteria are summarized. All fetal MRI performed between December 2019 and June 2021 were reviewed. Some examinations included sequences performed both in axial and coronal planes

All consecutive fetal MRI studies acquired between December 2019 and June 2021 were reviewed for potential inclusion. Inclusion criteria were as follows: (1) GA between 19 and 39 gestational weeks; (2) singleton pregnancies; (3) availability of all three sequences, either in the axial or coronal plane, within the same study: T1-weighted fast field echo (T1-FFE), T1-weighted fast FLAIR (T1-FFLAIR), and T1-weighted fast FLAIR compressed sense (T1-FFLAIR-CS) sequence; and (4) subjects without brain pathologies. We included six subjects with isolated borderline ventriculomegaly (trigonal width up to 13 mm), and one subject with an arachnoid cyst without mass effect, as well as two subjects with suspicion of very minor bleeding, and one with periventricular cyst, but with unremarkable clinical and sonographic follow-up postpartum. Participants who underwent more than one fetal MRI study were included each time in the corresponding age group if the sequences were available. Exclusion criteria were as follows: (1) unknown gestational age, (2) inappropriate image quality due to either artifacts or fetal movements, and (3) lack of concomitant acquisition of all three sequences in at least one corresponding orthogonal plane. A flowchart diagram of included and excluded MRI examinations is shown in Fig. 1.

### MRI protocol

The fetal MRI studies were performed as previously described [5, 10, 11] on a 1.5-T MR (Ingenia, Philips Healthcare) scanner using a body phase-array coil with six elements wrapped around the mother’s abdomen. The following three T1-weighted sequences for the fetal neuroimaging protocol were acquired: standard T1-weighted fast field echo (T1-FFE), as well as single-shot high-resolution TSE**-**based T1-weighted fast FLAIR (T1-FFLAIR), and T1-weighted fast FLAIR compressed sense (T1-FFLAIR-CS) sequences, either in the axial or coronal plane. Sequence parameters for T1-FFE, T1-FFLAIR, and T1-FFLAIR-CS are provided in Table 1.

**Table 1:** Acquisition parameters for the T1-weighted sequences performed for fetal MRI brain imaging

Parameter	T1-FFLAIR	T1-FFLAIR-CS	T1-FFE
Breath-hold	No	No	Yes
Echo time (ms)	3.9	4.4	4.6
Repetition time (ms)	10,000	10,000	131
Inversion time (ms)	1600	1600	no
Field of view (mm)	260 × 260 × 75	280 × 280 × 96	327 × 327 × 63
Acquisition voxel size (mm)	1.75 × 1.91 × 4	1.35 × 1.93 × 4	1.76 × 1.97 × 4
Reconstruction voxel size (mm)	1.02 × 1.02 × 4	0.97 × 0.97 × 4	1.14 × 1.14 × 4
Section thickness (mm)	4	4	4
Sensitivity-encoding factor	No	2.5	No
Half Fourier	No	No	No
Total acquisition time (s)	30	20	15

*T1-FFLAIR*, T1-weighted fast FLAIR; *T1-FFLAIR-CS*, T1-weighted fast FLAIR compressed sense; *T1-FFE*, T1-weighted fast field echo sequence

### Qualitative MRI analysis

Images were independently reviewed by two radiologists (RI.M. and V.S.) with 12 and 3 years of experience, respectively, in fetal brain MRI, who were blinded to any fetal information, including referral diagnosis and gestational age. On each of the three T1-weighted sequences, the signal intensity of five brain regions (medulla oblongata; pons; mesencephalon; thalamus; and central region—cortical and subcortical white matter) was evaluated. The signal intensity of the deep white matter of the occipital or frontal lobe [2, 12] was used as reference, because it was proved that these regions become myelinated after birth [1, 2, 13]. Myelination was visually rated according to a 5-point grading scale based on an existing myelination total score (MTS) [14]: the signal of the respective brain region was 0 (hypointense or isointense); 1 (slightly hyperintense); 2 (hyperintense); 3 (clearly hyperintense); or 4 (considerably hyperintense) compared to the signal intensity of the reference. Subsequently, the values of the individual brain regions were summed, thus resulting in a final MTS for each sequence. Myelin assessment was performed in the right hemisphere by default. In the case of inappropriate image quality of the right hemisphere, myelin assessment was performed in the left hemisphere. The two readers maintained a 2-week break between the evaluation of MTS on each of the T1-weighted sequences.

### Quantitative MRI analysis

One reader (RI.M.) performed region-of-interest (ROI) analysis in the studies with available T1-weighted sequences in the axial plane. The coronal plane was not used for quantitative analysis because of the potential inconsistency due to the size and the longitudinal orientation of the anatomical structures assessed.

ROIs that delineated the same five brain anatomic regions were placed manually (Supplementary Fig. 1) and the average signal intensities were calculated when the identification of anatomical regions presented sufficient image quality to enable the measurements (Fig. 1). In addition, at the level of the pons, ROIs delineating the tegmentum pontis and basis pontis were also calculated as in Schmidbauer et al [15] (Supplementary Fig. 1). This process was repeated for each of the three sequences, with the selection of equivalent sections in the same fetus. To compensate for differences in the signal intensities between distinctive MRI studies, we used as reference the signal intensity of the muscle (circular ROIs between 10 and 20 mm^2^), measured at the abdominal wall, which was not influenced by the gestational age (see Supplementary Fig. 2). The signal intensity measured at each anatomic site was divided by the signal intensity of the muscle, thus resulting signal intensity (SI) ratios. The quantitative analysis was performed only by one reader as the studies of Schmidbauer et al [16, 17] showed a good agreement between readers when performing quantitative measurements at similar anatomical sites.

### Statistical analysis

The statistical analysis was performed by M.W. using R version 4.0.5 (https://www.r-project.org/). Continuous variables were analyzed using means and standard deviations. To assess the correlations between MTS, as well as signal intensity ratios and GA, Pearson’s correlation analysis was performed. Pearson and Filon’s *z* test were used to assess whether the correlations of MTS and signal intensity ratios with GA were significantly different between sequences. For this purpose, the values of MTS assessed per patient, sequence, and orthogonal plane were averaged across the two raters. Inter-rater reliability of the MTS assessment between the two readers was calculated using the intraclass correlation coefficient (ICC). ICC was calculated over all included subjects using two-way random effects model and a “single rater” unit, using the psych R package. Additionally, to determine the reproducibility of MTS ratings across axial and coronal sequences, ratings were averaged across readers and ICC between mean MTS rating scores for axial and coronal sequences were calculated for subjects in which both axial and coronal images were available. Two-way random effects models (ICC2) were used. Poor reliability was defined as an ICC value of less than 0.50, moderate reliability as an ICC of 0.50–0.75, good reliability as an ICC of 0.75–0.89, and excellent reliability as an ICC of 0.90 or higher [18, 19]. The Wilcoxon rank sum test was used for comparison of MTS and SI ratios among different GA.

### Histological analysis

The use of the postmortem brain tissue was approved by the Ethics Committee of the School of Medicine, University of Zagreb (MFSZ 380–59-1016–19-111/210 to NJM), and the Ethics Committee of University Hospital Centre Zagreb (KLASA: 8.1–17/142–2. No.02/21AG to NJM). The fetal brains (in total 46, staged from GA 18 to 42 weeks [20]) were conserved by immersion in 10% buffered formaldehyde. Brain blocks were gradually dehydrated in ethanol (70%, 80%, 96%, and 100%), cleared by toluene, paraffined (at 60 °C), cooled at 4 °C, and serially cut into 20-µm-thin sections on a sliding microtome. Finally, indirect immunohistochemistry staining was performed according to the protocol described previously [21]. Briefly, after dewaxing with xylol, sections were rehydrated in a graded series of ethanol solutions, washed 3 × 10 min with phosphate-buffered saline, immersed in citrate buffer (pH 6.0), and heated in a microwave for 15 min at 90 °C for antigen retrieval. After washing, the sections were immersed in 0.3% hydrogen peroxide (in a 3:1 mixture of methanol and re-distilled water) for 30 min, washed in phosphate-buffered saline, and then incubated for 2 h in the blocking solution (phosphate-buffered saline containing 5% bovine serum albumin and 0.5% Triton X-100, all from Sigma) at room temperature (RT) to prevent non-specific background staining. The incubation with primary antibody mouse anti-myelin basic protein (1:1000; MBP; clone SMI-99, SMI-99P; Covance) lasted for 48 h at 4 °C; sections were then rinsed in phosphate-buffered saline, and further incubated with secondary antibody anti-mouse (Vectastain ABC kit, PK 4010) for 1 h at RT (Vectastain ABC kit; Vector Laboratories). Vectastain ABC reagents (streptavidin–peroxidase complex) were used in the subsequent step for 1 h at RT, and rinsed in phosphate-buffered saline for 3 × 10 min, and finally, peroxidase activity was visualized with 3,3′-diaminobenzidine peroxidase substrate (Sigma-Aldrich). The sections were then rinsed with phosphate-buffered saline, dried, cleared in Polyclear, and cover-slipped with PolyMount (Polysciences, Inc.). The negative controls were included in all immunohistochemical experiments by (1) replacing the primary antibody with a blocking solution, or by (2) omitting the secondary antibody or replacing it with an inappropriate secondary antibody. The histological sections were analyzed using an Olympus BX53 microscope, and images were obtained using a high-resolution digital slide scanner NanoZoomer 2.0RS (Hamamatsu).

## Results

### Study cohort

The study cohort comprised 115 MRI studies performed on 108 pregnant women (seven women received two examinations) with a mean GA of 28.56 ± 5.23 weeks (range 19–39 weeks). The distribution of included fetuses according to gestational age is provided in Supplementary Table 1. For the qualitative MRI analysis, we identified 76 examinations performed in the coronal plane and 63 examinations performed in an axial plane which fulfilled the inclusion criteria. Sixty-one of the studies completed in the axial plane were available for quantitative analysis. Supplementary Table 2 summarizes the number of cases at each anatomical brain region in which the images presented sufficient image quality to enable quantitative measurements.

### Qualitative analysis

**Fig. 2:**
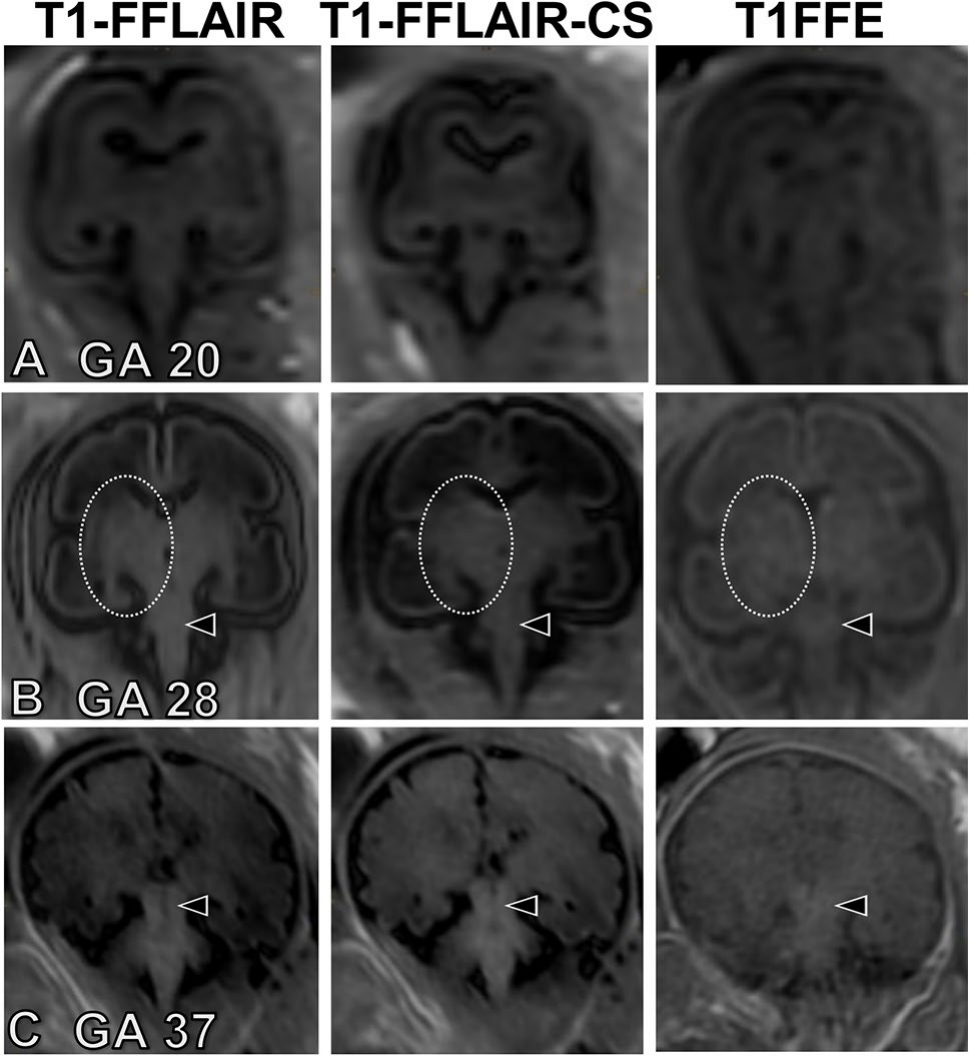
Coronal T1-weighted fast fluid-attenuated inversion-recovery (T1-FFLAIR), T1-weighted fast fluid-attenuated inversion-recovery with compressed sensing (T1-FFLAIR-CS), and T1-fast field echo sequence (T1-FFE) images at gestational age (GA) 20 (**A**), 28 (**B**), and 37 (**C**) showing the increase in signal intensity at the level of the brainstem and thalamus during fetal development, indicating progressive myelination. Note that the signal intensity of the brainstem (*arrowheads*) and thalamus (*circles*) is higher on T1-FFLAIR and T1-FFLAIR-CS compared to T1-FFE

**Fig. 3:**
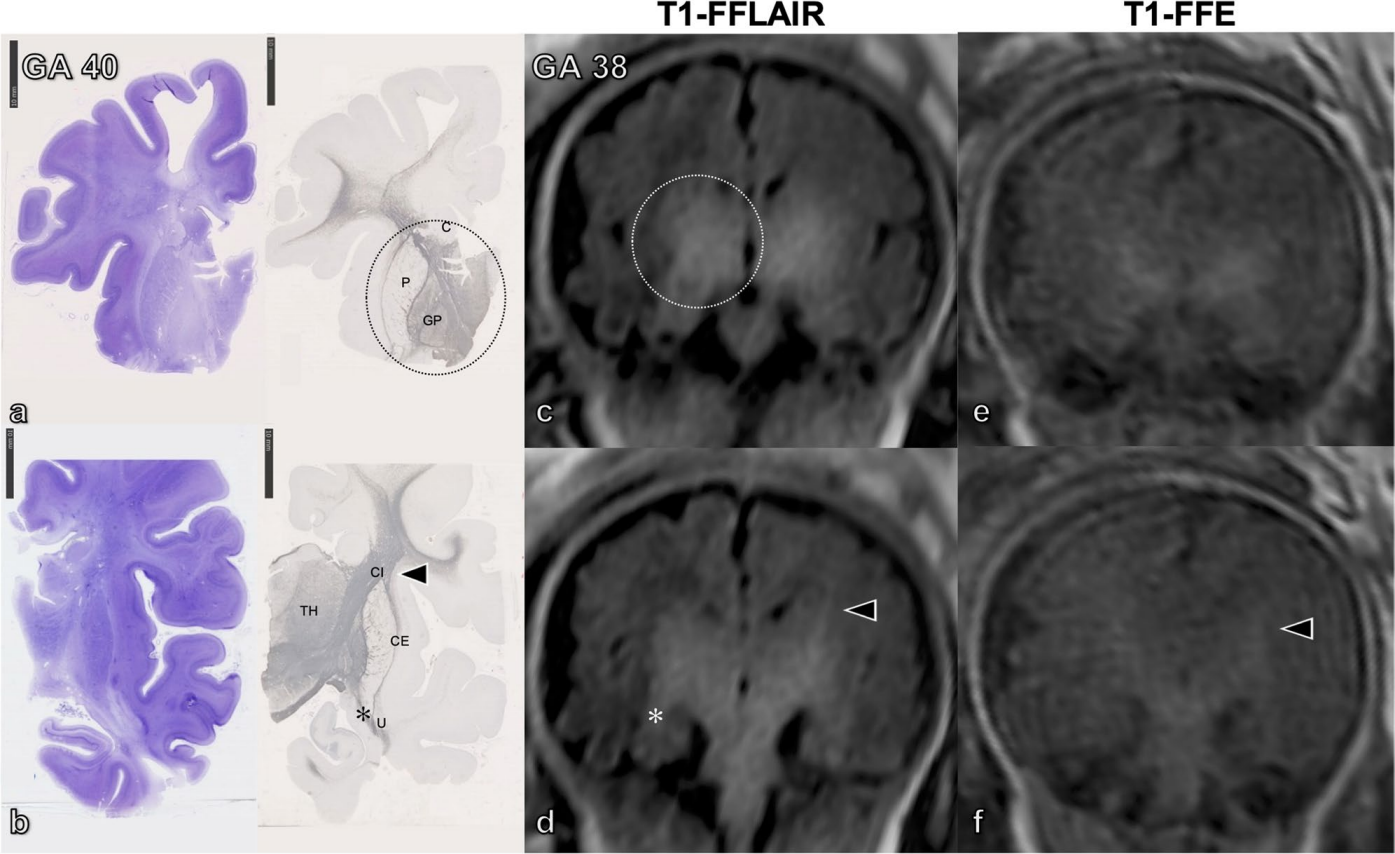
**a**, **b** Histological sections (coronal) of postmortem fetal human brain at gestational age (GA) 40 stained with cresyl violet (**a** and **b**, left) and myelin basic protein (MBP) (**a** and **b,** right). The images show the myelination process, which at this GA extends to the entire thalamus (TH), forebrain basal ganglia (except the putamen, P, and caudatus, C), the capsula interna (CI, *arrowheads*), and the capsula externa (CE), the uncinated fasciculus (U, *asterisk*), most of the thalamic nuclei, and the subthalamic region, as well as the cerebral peduncles. Corresponding coronal T1-weighted fast fluid-attenuated inversion-recovery (T1-FFLAIR) (**c** and **d**) and T1-fast field echo sequence (T1-FFE) (**e** and **f**) in utero images of another fetus at GA 38 depicting a significantly better delineation of the myelinated structures on the T1-FFLAIR sequence than on T1-FFE

**Fig. 4:**
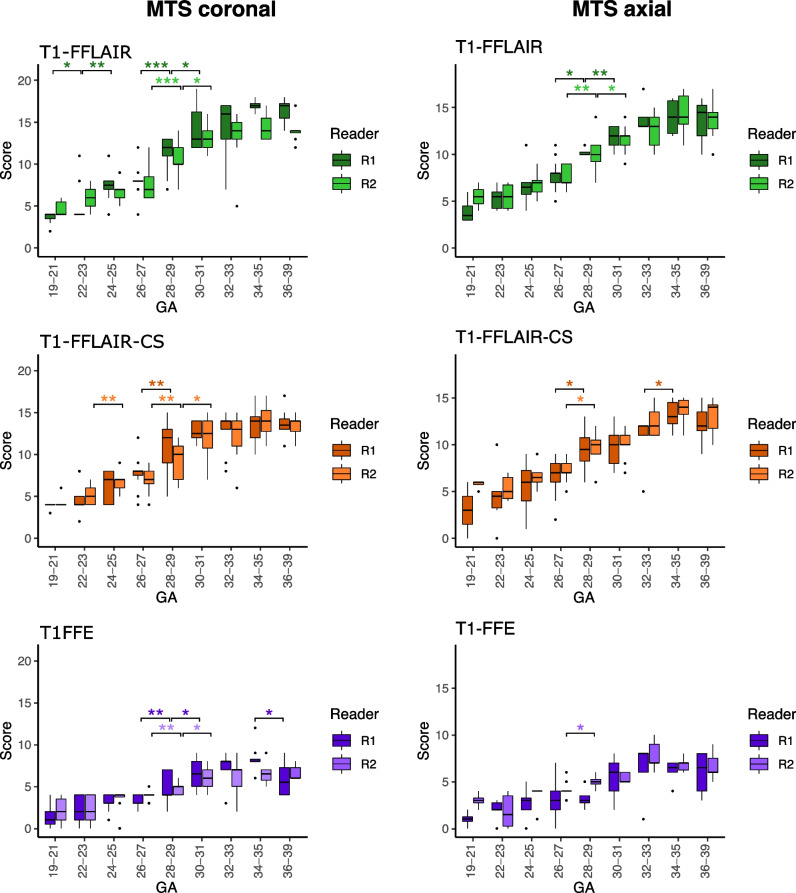
Summary of the qualitative analysis. Boxplots showing the dynamic of myelin total score (MTS) at the corresponding gestational age on each of the three T1-weighted sequences. Note the larger increase in MTS on T1-weighted fast fluid-attenuated inversion-recovery (T1-FFLAIR) and T1-weighted fast fluid-attenuated inversion-recovery with compressed sensing (T1-FFLAIR-CS) sequences compared to T1-fast field echo sequence (T1-FFE) during fetal development (see also the calculated slopes for the qualitative and quantitative analysis in Supplementary Table 3). ***p < 0.001; **p < 0.01; *p < 0.05

**Fig. 5:**
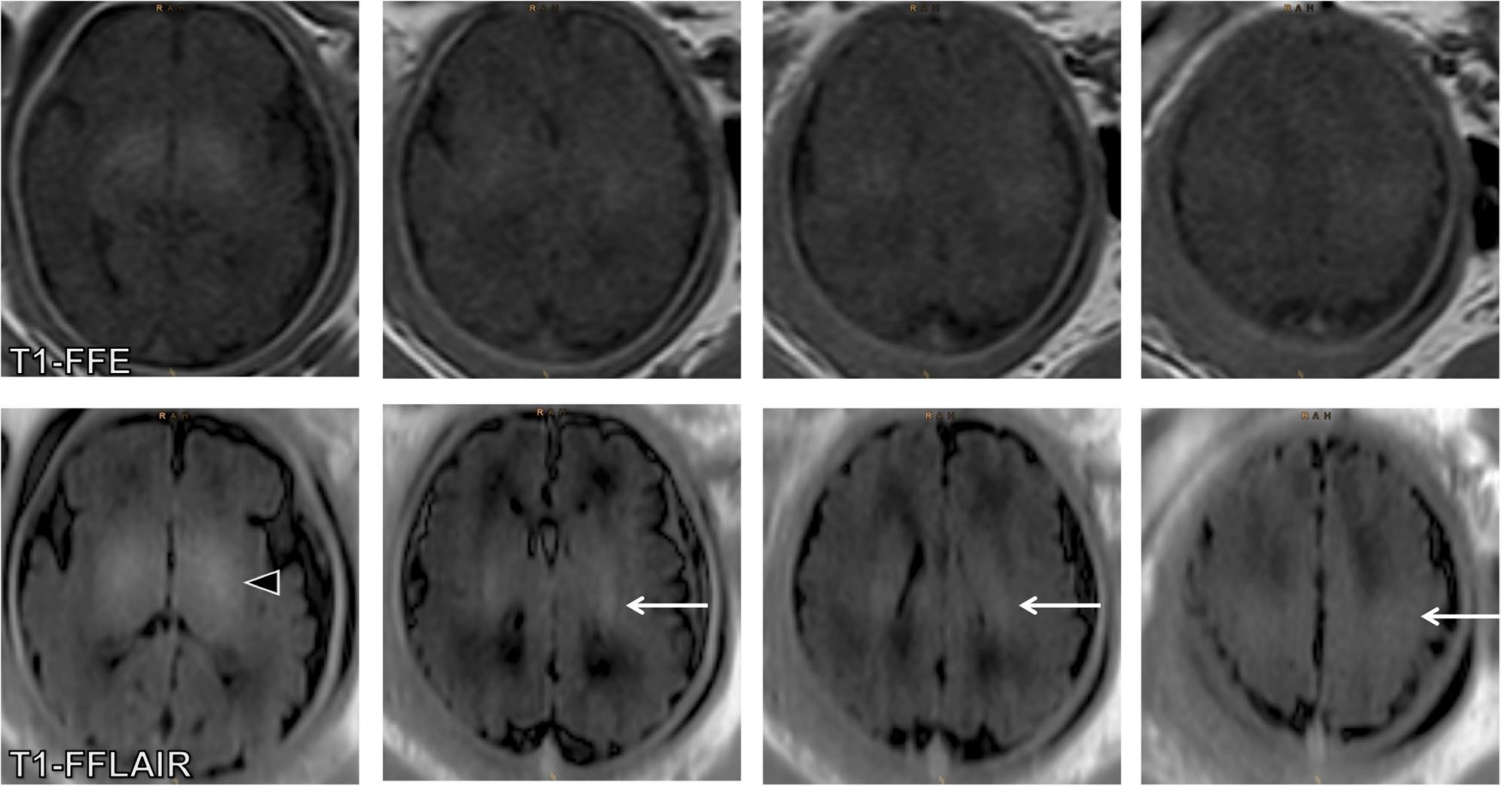
Axial images from T1-fast field echo sequence (T1-FFE) (*upper row*) and T1-weighted fast fluid-attenuated inversion-recovery (T1-FFLAIR) (*lower row*) sequences at gestational age (GA) 37, showing the clearly hyperintense signal in the globus pallidus (*arrowhead*), as well as the corona radiata and the central region (*arrows*) on the T1-FFLAIR sequence. On the T1-FFE sequence, the signal of these structures is only slightly hyperintense and the corona radiata is only partially identified

The signal intensity of white matter in the brainstem, thalamus, and central region was low at early examined GA and increased with maturation during fetal development (Fig. 2). Overall, a myelination-corresponding increase in signal intensity was seen best on the T1-FFLAIR sequence (Figs. 2, 3, and 4). Furthermore, when compared with the histological sections, the hyperintense signal on the MRI sequences correlated well with the myelinated anatomical sites (Fig. 3; Supplementary Figs. 3 and 4), with the highest signal intensity demonstrated on T1-FFLAIR. Also, the identification and visualization of smaller myelinated anatomic structures, such as the globus pallidus, capsula interna, or corona radiata, were superior on the T1-FFLAIR sequence (Fig. 3 and Fig. 5).

The fetal MTS positively correlated with GA on all three sequences (Fig. 4 and Table 2, *r* = 0.908 for T1-FFLAIR; *r* = 0.886 for T1-FFLAIR-CS; and *r* = 0.829 for T1-FFE for the coronal plane and *r* = 0.888 for T1-FFLAIR; *r* = 0.861 for T1-FFLAIR-CS; and *r* = 0.802 for T1-FFE for the axial plane). The correlation of fetal MTS with GA was significantly better on T1-FFLAIR sequence, both in the coronal and axial plane, when compared to T1-FFE (Table 2, all *p* < 0.01). MTS increased significantly at GA 28–29 on all three sequences, both on axial and coronal planes (Fig. 4). There was a high degree of concordance in MTS assessment by both raters, both in the coronal and axial planes (Table 3). The inter-rater reliability was good to excellent for T1-FFLAIR (ICC of 0.856 in the coronal plane and of 0.863 in the axial plane); good for T1-FFLAIR-CS (ICC of 0.843 in the coronal plane and of 0.764 in the axial plane); and only moderate on T1-FFE (ICC of 0.650 in the coronal plane and of 0.525 in the axial plane). All three sequences showed excellent reproducibility across axial and coronal planes in fetuses for which both axial and coronal images were available (*n* = 33), with T1-FFLAIR showing an ICC of 0.92 (95% CI 0.79–0.96), T1-FFLAIR-CS an ICC of 0.92 (95% CI 0.82–0.96), and T1-FFE an ICC of 0.89 (95% CI 0.81–0.94).

**Table 2:** Pearson correlation coefficients of the T1-weighted sequences with the gestational age

Parameter	T1-FFLAIR	T1-FFLAIR-CS	T1-FFE	*p*^a^	*p*^b^	*p*^c^
Qualitative analysis						
MTS (axial plane)	0.888	0.861	0.802	0.249	0.006	0.080
MTS (coronal plane)	0.908	0.886	0.829	0.137	0.001	0.018
Quantitative analysis						
Medulla oblongata	0.678	0.340	0.424	0.001	0.044	0.581
Pons	0.624	0.443	0.312	0.078	0.022	0.400
Mesencephalon	0.764	0.276	0.385	< 0.001	0.001	0.467
Thalamus	0.583	0.357	0.240	0.032	0.007	0.415
Central region	0.786	0.448	0.425	< 0.001	< 0.001	0.850

*T1-FFLAIR*, T1-weighted fast FLAIR; *T1-FFLAIR-CS*, T1-weighted fast FLAIR compressed sense; *T1-FFE*, T1-weighted fast field echo sequence

*p*^a^—*p* value for the comparison between T1-FFLAIR and T1-FFLAIR-CS; *p*^b^—*p* value for the comparison between T1-FFLAIR and T1-FFE; *p*^c^—*p* value for the comparison between T1-FFLAIR-CS and T1-FFE

**Table 3:** The inter-rater reliability of the myelin total score

Sequence	ICC (95% CI) Coronal plane	ICC (95% CI) Axial plane
T1-FFLAIR	0.856 (0.794–0.899)	0.863 (0.799–0.908)
T1-FFLAIR-CS	0.843 (0.786–0.885)	0.764 (0.649–0.842)
T1-FFE	0.650 (0.542–0.737)	0.525 (0.335–0.669)

*ICC*, intraclass correlation coefficients; *CI*, confidence interval; *T1-FFLAIR*, T1-weighted fast FLAIR; *T1-FFLAIR-CS*, T1-weighted fast FLAIR with compressed sensing; *T1-FFE*, T1-weighted fast field echo sequence

### Quantitative analysis

**Fig. 6:**
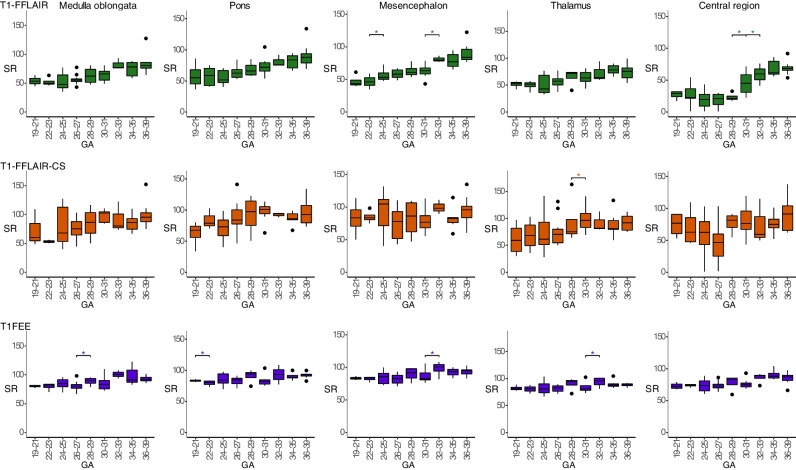
Summary of the quantitative analysis. Boxplots presenting the results of the quantitative analysis performed at the levels of the medulla oblongata, pons, mesencephalon, thalamus, and central region on each of the three T1-weighted sequences. Note the better increase in signal intensity ratios (SI) on T1-weighted fast fluid-attenuated inversion-recovery sequence (T1-FFLAIR) and the larger variability in the measurements on T1-weighted fast fluid-attenuated inversion-recovery with compressed sensing sequence (T1-FFLAIR-CS) sequences due to the signal inhomogeneity. ***p < 0.001; **p < 0.01; *p < 0.05

The results of the quantitative analysis are presented in Fig. 6 and Table 2. The signal intensity ratios measured at the five brain regions correlated best with GA on T1-FFLAIR (*r* between 0.583 and 0.785). T1-FFLAIR demonstrated significantly better correlations with GA than T1-FFE for both qualitative and quantitative analysis (all *p* < 0.05). On all three sequences, within the same subject, the ratio between SI of the tegmentum pontis and basis pontis was higher than 1 (median for all GA and sequences between 1. 0 and 1.26). Furthermore, the SI of the tegmentum pontis showed steeper slopes (Supplementary Table 3) during fetal development than the SI of basis pontis, thus demonstrating the ventral to dorsal gradient in myelination at the level of the pons.

## Discussion

In this study, the T1-weighted fast FLAIR sequence enabled a better visualization and assessment of prenatal brain myelination when compared with standard used T1 fast field echo sequence, as determined by qualitative and quantitative analysis of MRI images with histological correlation.

Myelination begins prenatally and progresses systematically from caudal (spinal cord) to rostral parts (brain) and spreads from the central (diencephalon, pre- and postcentral gyri) to peripheral parts of the brain [22], following the development of afferent and efferent cortical connections [23]. Before myelination is initiated, oligodendrocyte precursors transform first into premyelinating oligodendrocytes and then into mature myelin-producing cells [24]. Premyelinating oligodendrocytes are present already at the beginning of mid-gestation (GA 17–20), while the onset of myelination at different anatomical sites has been described to occur later during the last trimester [24–26]. On histology, the first signs of myelination appear in the spine at GA 16, in the cerebellar tracts at GA 20, in the globus pallidus and thalamus at GA 20–25, in the pyramidal tracts at GA 36, in the precentral and postcentral gyri at GA 35, and in the anterior internal capsule and optic radiation at GA 35–37 [1, 27, 28].

MRI enables the visualization of myelination progress in vivo. A hyperintense T1 signal occurs in the same topographical and temporal sequence as myelination progresses, due to the increase in cholesterol and glycolipid content that accompanies the formation of myelin [2].

This study assessed the visualization and progression of myelination in several regions of the brain, and showed that the myelin total score on all evaluated sequences correlated well with the GA. MTS showed a continuous progressive increase in value, suggesting the accumulation of myelin with increasing GA. These findings are concordant with published MRI data about the time-course of onset and progression of myelination in preterm neonates [3, 4], and with histopathological reports [28]. The dynamic range of MTS (Supplementary Table 3) was highest on the T1FFLAIR sequence, correlating with previous results showing that T1-weighted inversion-recovery sequences provide increased tissue contrast and are less sensitive to motion and CSF flow-pulsation artifacts in the adult brain [7, 29] and spine [8]. A similar T1-weighted inversion-recovery sequence was shown to enable superior diagnostic quality and increased dynamic range of contrast compared to the standard T1-weighted gradient-echo protocol on fetal MRI [6]. Overall, the quantitative analysis showed inferior correlation coefficients of SI ratios with GA compared to MTS on all sequences, most likely because MTS sums up and integrates the changes in several anatomical locations, compared to the changes at these individual locations taken separately.

Myelination is an important process during brain maturation and its impairment is associated with neurodevelopmental disorders [30]. While assessment of myelination is done routinely in postnatal MR scans for prognostic purposes, especially in preterms [3, 4], this could not be done sufficiently prenatally due to the lack of a suitably myelin-sensitive MR sequence. This study showed that myelination in utero was best visualized on the T1-FFLAIR sequence, in both the axial and coronal planes. Furthermore, we observed that MTS increased significantly at GA 28–29 and the signal intensity ratios on the T1-FFLAIR sequence in the central region started to increase at GA 30, earlier than previously reported. In the work of Abe et al [12], the longitudinal changes in the signal intensity ratios measured on a T2-weighted sequence, corresponding to the time-course of progression of myelination, generally appeared after GA 32. The results of our study could be attributable to the superior tissue contrast on T1-FFLAIR and to the T1-weighted signal changes, which start already during the “premyelinating” state [31] and have been shown to precede those in T2-weighted sequences [2, 3]. T1-FFLAIR was superior in depicting myelination at individual anatomic locations, such as the corona radiata or the central region, when compared to T1-FFE.

One advantage of T1-FFLAIR is that it does not require breath-holding, such as T1-FFE, and, hence, is better tolerated by pregnant women. We attempted to decrease the acquisition duration using the compressed sensing technique, which acquires k-space in an undersampled fashion and aims to reconstruct MRI images from much fewer encoding steps [32, 33]. The result is a reduced scan time without sacrificing image quality. Currently, CS has been employed in many applications in adult [9, 34] and pediatric [35, 36] MRI. The inferior results calculated on T1-FFLAIR-CS sequence, when compared with T1-FFLAIR, can be explained through the larger variation in the calculated values of SI due to signal inhomogeneity (Fig. 6; Supplementary Fig. 5), most probably because of the undersampling of k-space. Although T1-FFLAIR-CS demonstrated an increased inhomogeneity of the signal intensity, it presented good structural details and the diagnostic findings visually (MTS) were similar between conventional and CS sequences.

Prenatal assessment of myelination should be done, as impairment of the myelinating processes may start early in diseases known or suspected to be associated with prenatal myelination deficits, such as iron deficiency [37], fetal alcohol spectrum disorders [38], opioids [39], Alexander disease type I, vanishing white matter disease, or hypomyelination of early myelinating structures (HEMS) [40]. Using the T1-FFLAIR sequence, even quantitative assessment of the premyelinating process has become feasible prenatally.

Previous in vivo MRI studies were acquired in preterm brains, but prematurity can delay regular brain myelination [14]. This study was performed under normal fetal development settings, both qualitatively and quantitatively, and MRI images were compared with histology as gold standard. ICC yielded a good correlation between raters for both T1-FFLAIR and T1-FFLAIR-CS, showing that these measurements are reproducible, reliable, and superior to T1-FFE in assessing fetal myelination.

There were several limitations to our study. First, this was a single-center retrospective study. Second, we included ten subjects with suspicion of minor pathologies, but their results did not differ from the results of the fetuses at equivalent GA. Third, additional small anatomic sites with evidence of myelination before birth, such as the cerebellar vermis, dentate nucleus, posterior internal capsule, or optic radiation, were not assessed because these structures are not reliably identifiable and/or evaluable at earlier gestational ages. Fourth, MRI signal intensity measurements, particularly in fetal MRI, are inherently variable and challenging to reproduce, as distance to the coil or field inhomogeneities cannot be controlled. We tried to minimize this variability by referencing the signal intensity of the abdominal muscle for the quantitative analysis, which we showed to remain fairly constant during gestation.

## Conclusions

This study demonstrated that T1-FFLAIR sequence outperforms the standard T1-FFE sequence in the visualization of fetal brain myelination. Given these advantages and the relatively short acquisition time, we recommend its use as part of the routine fetal brain examination.

### Supplementary information

Below is the link to the electronic supplementary material.

Supplementary file1 (PDF 433 KB)

## Abbreviations

CNS: Central nervous system
CS: Compressed sensing technique
CSF: Cerebrospinal fluid
GA: Gestational age
ICC: Intraclass correlation coefficient
MBP: Myelin basic protein
MRI: Magnetic resonance imaging
MTS: Myelin total score
ROI: Region-of-interest
RT: Room temperature
SI: Signal intensity ratios
T1-FFE: T1 fast field echo
T1-FFLAIR: Fast fluid-attenuated inversion-recovery
T1-FFLAIR-CS: T1-weighted fast fluid-attenuated inversion-recovery compressed sense

## Declarations

### Guarantor

The scientific guarantor of this publication is Ruxandra-Iulia Milos.

### Statistics and biometry

Martin L. Watzenboek has significant statistical expertise and provided the statistical analysis for this manuscript.

No complex statistical methods were necessary for this paper.

### Methodology

• retrospective study

• observational

• performed at one institution
