# Supplementary material for: T1-weighted fast fluid-attenuated inversion-recovery sequence (T1-FFLAIR) enables the visualization and quantification of fetal brain myelination in utero
Source: Eur Radiol. 2023 Nov 29;34(7):4573–84. doi: 10.1007/s00330-023-10401-z (PMC11213743; doi:10.1007/s00330-023-10401-z)

T1-weighted fast fluid-attenuated inversion-recovery sequence (T1-FFLAIR) enables the visualization and quantification of fetal brain myelination *in utero*

Electronic Supplementary Material (ESM)

Eur Radiol (2023) Milos RI, Schmidbauer V, Watzenboeck ML et al.

Supplementary material

Supplementary Table 1: Distribution of included fetuses according to gestational age

Gestational age	Number of cases	
	Qualitative analysis<br>Coronal plane	Qualitative analysis<br>Axial plane
19-21	5	4
22-23	8	6
24-25	10	8
26-27	11	13
28-29	10	4
30-31	9	9
32-33	7	5
34-35	8	6
36-39	8	8

Supplementary Table 2: Number of cases at each anatomical brain region in which the images presented sufficient image quality to enable quantitative measurements

**Anatomical region**	**T1-FFLAIR**	**T1-FFLAIR-CS**	**T1-FFE**
Medulla oblongata	55	52	49
Pons	57	54	54
Mesencephalon	58	56	57
Thalamus	58	56	56
Central region	58	56	57

Supplementary Table 3: Calculated slopes for the increase in MTS and for the signal intensity ratios demonstrating the development of myelination during the fetal period. Note the higher dynamic range/slope values for T1-FFLAIR and T1-FFLAIR-CS sequences when compared to standard T1-FFE sequence. Furthermore, higher slope values were calculated for tegmentum pontis when compared to basis pontis for the same sequence, thus demonstrating a gradient in myelination in these areas.

**Slopes**			
	**T1-FFLAIR**	**T1-FFLAIR-CS**	**T1-FFE**
**MTS coronal (R1/R2)**	0.88/0.67	0.68/0.65	0.39/0.32
**MTS axial (R1/R2)**	0.66/0.64	0.59/0.58	0.34/0.31
**Anatomical region**			
Medulla oblongata	2.4	1.82	0.94
Pons (basis pontis und tegmentum pontis)	2.7	1.79	0.61
Basis pontis	1.98	1.06	0.48
Tegmentum pontis	2.7	1.7	1.13
Mesencephalon	2.7	1.2	0.83
Thalamus	2.12	1.5	0.56
Central region	3.30	2.08	0.99

Supplementary Fig. 1: Placement of regions of interest for the quantitative analysis demonstrated on a T1-fast field echo sequence

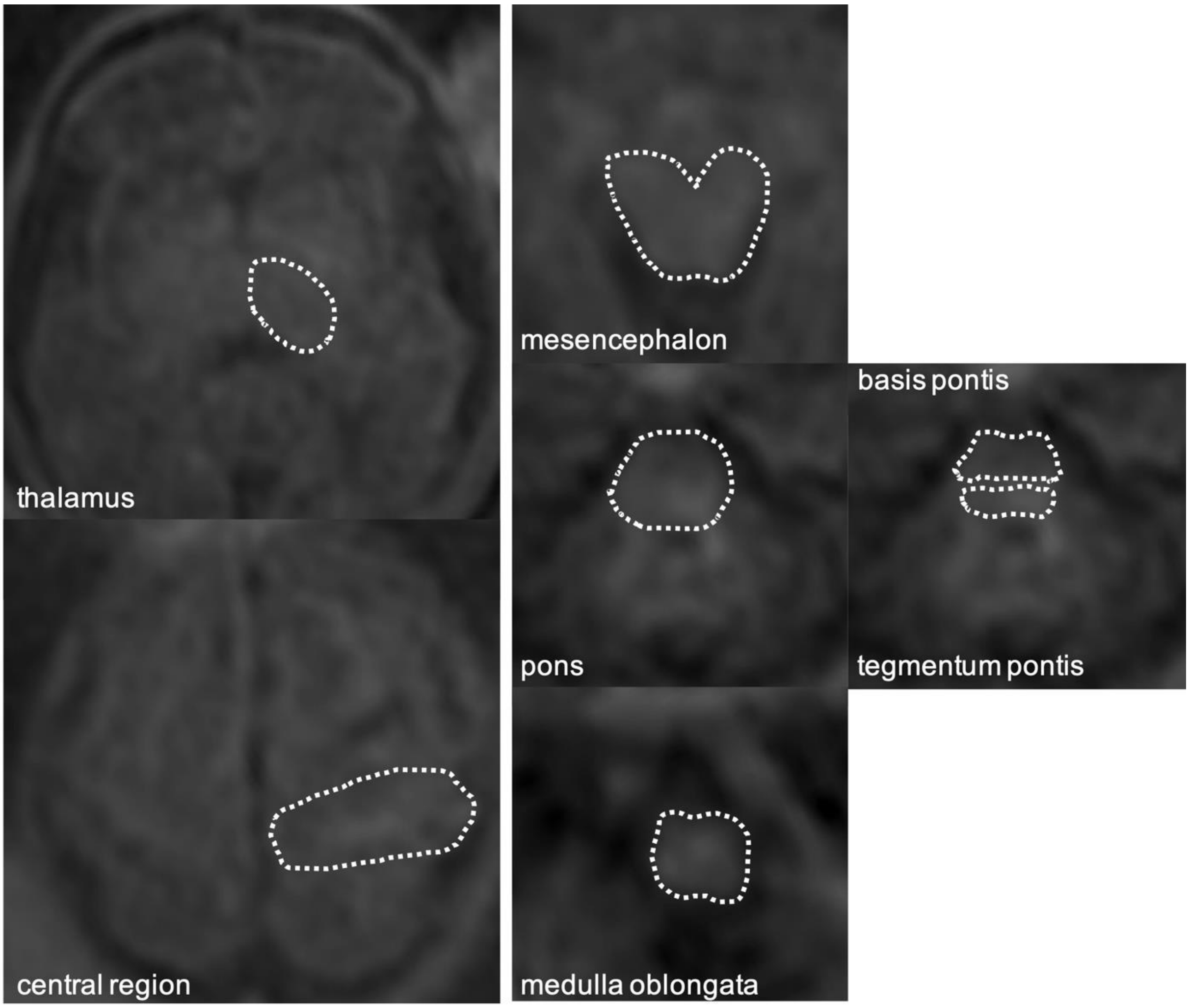

Supplementary Fig. 2: Boxplots showing the signal intensity of the muscle during fetal development, measured at the abdominal wall, indicating that it was not influenced by gestational age. T1-FFLAIR, T1-weighted fast fluid-attenuated inversion-recovery; T1-FFLAIR-CS, T1-weighted fast fluid-attenuated inversion-recovery with compressed sensing; T1-FFE, T1-fast field echo sequence

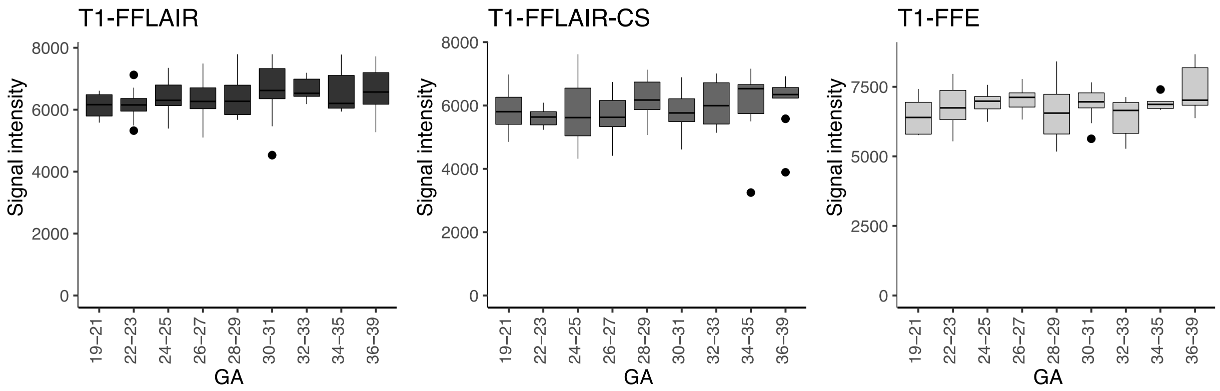

Supplementary Fig 3: (**a** and **b**) Histological sections of postmortem fetal human brain at gestational age (GA) 19 (**a**) and GA 42 (**b**) stained immunohistochemically for myelin basic protein (MBP). The MBP expressed by the oligodendrocytes demarcates the spatial-temporal sequence of prenatal myelination of axonal tracts and fibers. The onset of myelination of the medial longitudinal fasciculus at GA 19 (**a**); myelination presented with MBP labeled almost all fibers in the pons and the tegmentum at an age equivalent to GA 42. Axial T1-weighted fast fluid-attenuated inversion-recovery (T1-FFLAIR) (**c** and **d**) and T1-fast field echo sequence (T1FFE) (**e** and **f**) fetal images at GA 20 (**c** and **e**) and GA 39 (**d** and **f**) at locations corresponding to the histological images, showing similar progression of brainstem myelination during fetal development *in utero*. Note the clear hyperintense signal on T1-FFLAIR image when compared to T1-FFE image at the later developmental age (*arrowheads*).

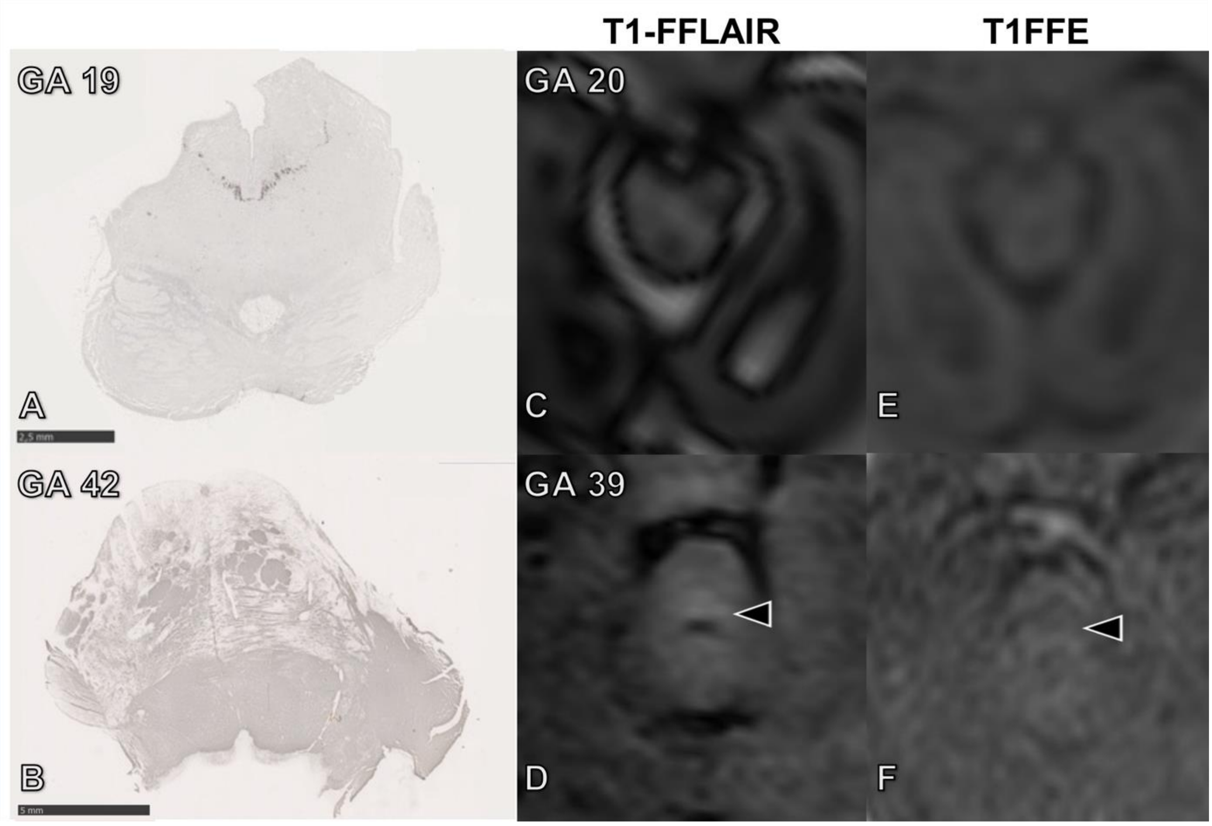

Supplementary Fig. 4: (**a** and **b**) Histological sections (coronal) of postmortem fetal human brain at gestational age (GA) 33 stained with Cresyl-violet (modification by Nissl), (**a** and **b**, left) or immunohistochemically for myelin basic protein (MBP) (**a** and **b,** right). The beginning of myelination in the anterior parts of the thalamus, in the basal forebrain ganglia (**a**) and the pretectum, as well as in the intermediate and subplate zone (future white matter, *arrowhead*, **b**) of the paracentral and parietal lobe is evident. Some fibers in the nucleus reticularis thalami display MBP; then, fibers of the zona incerta (ZI) and subthalamic nucleus (ST) show myelination to a certain degree. Laterally from the genu of the capsula interna (mainly negative for MBP staining), the globus pallidus (GP) pars medialis and lateralis, as well as the lamina medullaris (medialis and lateralis) are myelinated (putamen, P, and caudatus, C, are negative for MBP staining). Also, clear MBP staining of some radial fibers from the basal forebrain toward the cortical plate is visible. Fine MBP staining of tangentially running basal forebrain fibers in the intermediate zone and some radially oriented in the subplate zone, elongating toward the dorsal regions of the future cortex are also visible***.*** Coronal T1-weighted fast fluid-attenuated inversion-recovery (T1-FFLAIR) (**c** and **d**) and T1-fast field echo sequence (T1FFE) (**e** and **f**) *in utero* images of another fetus at GA 33 at locations similar to the histological images (*circles*), with a clear hyperintense signal on T1-FFLAIR corresponding to the myelinated structures.

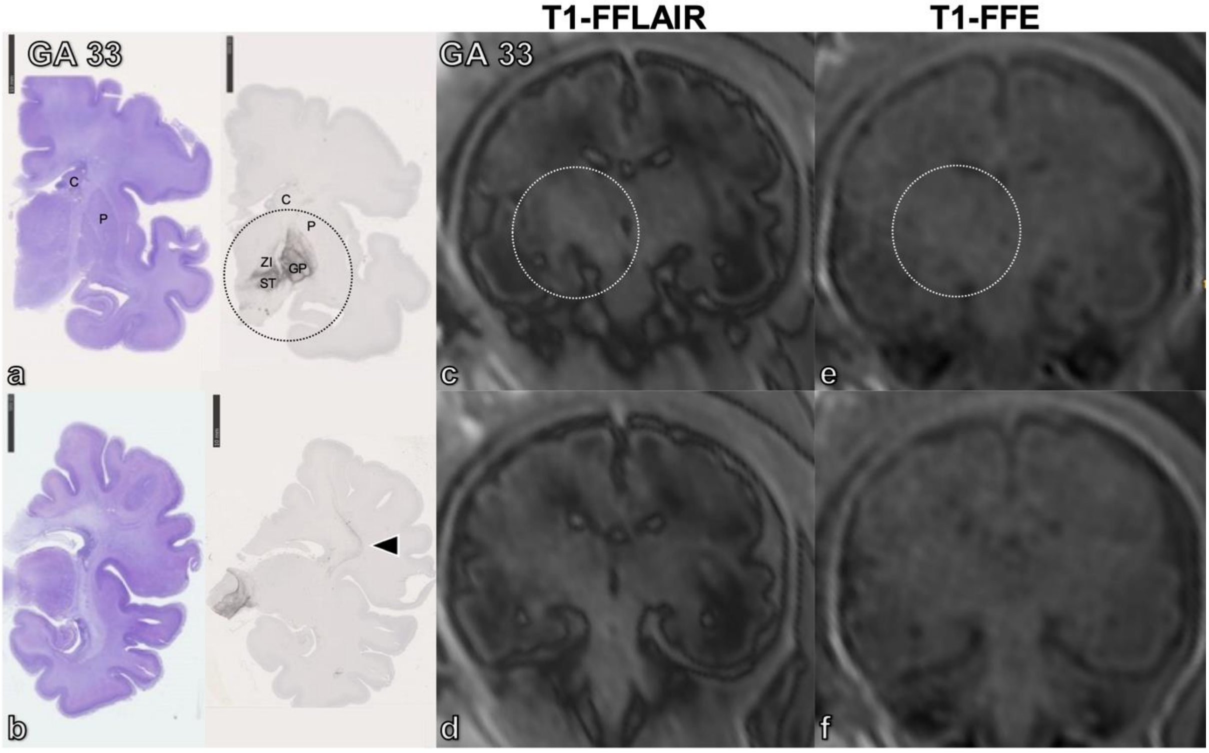
T1-FFLAIR
T1-FFE
GA 33
C
P
C
P
ZI
ST
GP
a
GA 33
c
e
b
d
f

Supplementary Fig. 5: Coronal consecutive images of a T1-weighted fast fluid-attenuated inversion-recovery with compressed sensing (T1-FFLAIR-CS) sequence in a fetus at gestational age of 26 weeks. There is a high inhomogeneity of the signal intensity of the cerebrospinal fluid (*arrows*), as well as the brain parenchyma, within the same sequence.

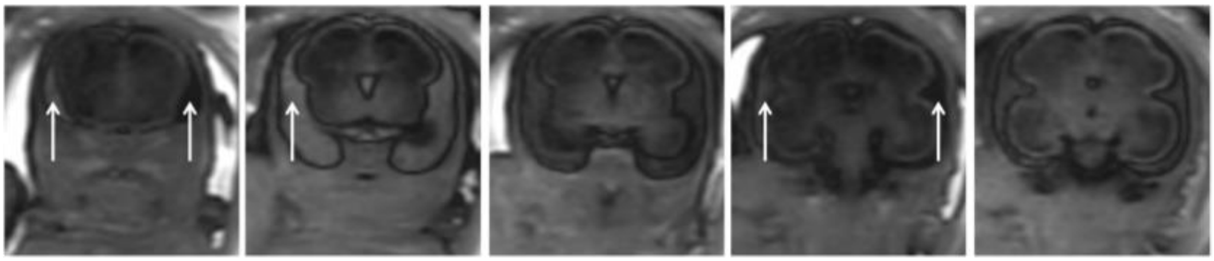

Supplement: Supplementary file 1 — Supplementary file1 (PDF 433 KB) [file 330_2023_10401_MOESM1_ESM.pdf]
